# Can CCTV identify people in public transit stations who are at risk of attempting suicide? An analysis of CCTV video recordings of attempters and a comparative investigation

**DOI:** 10.1186/s12889-016-3888-x

**Published:** 2016-12-15

**Authors:** Brian L. Mishara, Cécile Bardon, Serge Dupont

**Affiliations:** 1 Centre for Research and Intervention on Suicide and Euthanasia (CRISE), Psychology Department, Université du Québec à Montréal, CRISE-UQAM, C.P. 8888, Succ. Centre-ville, Montreal, QC H3C 3P8 Canada; 2 Metro Network, Société des Transports de Montréal, Montréal, Canada

**Keywords:** Metro, Suicide, Underground, Subway, Prevention, Public transit, CCTV, Risk

## Abstract

**Background:**

Suicides incur in all public transit systems which do not completely impede access to tracks. We conducted two studies to determine if we can reliably identify in stations people at risk of suicide in order to intervene in a timely manner. The first study analysed all CCTV recordings of suicide attempters in Montreal underground stations over 2 years to identify behaviours indicating suicide risk. The second study verified the potential of using those behaviours to discriminate attempters from other passengers in real time.

**Methods:**

*First study*: Trained observers watched CCTV video recordings of 60 attempters, with 2–3 independent observers coding seven easily observable behaviours and five behaviours requiring interpretation (e.g. “strange behaviours,” “anxious behaviour”). *Second study*: We randomly mixed 63 five-minute CCTV recordings before an attempt with 56 recordings from the same cameras at the same time of day, and day of week, but when no suicide attempt was to occur. Thirty-three undergraduate students after only 10 min of instructions watched the recordings and indicated if they observed each of 13 behaviours identified in the First Study.

**Results:**

*First study*: Fifty (83%) of attempters had easily observable behaviours potentially indicative of an impending attempt, and 37 (61%) had two or more of these behaviours. Forty-five (75%) had at least one behaviours requiring interpretation. Twenty-two witnesses attempted to intervene to stop the attempt, and 75% of attempters had behaviours indicating possible ambivalence (e.g. waiting for several trains to pass; trying to get out of the path of the train). *Second study*: Two behaviours, leaving an object on the platform and pacing back and forth from the yellow line (just before the edge of the platform), could identify 24% of attempters with no false positives. The other target behaviours were also present in non-attempters. However, having two or more of these behaviours indicated a likelihood of being at risk of attempting suicide.

**Conclusions:**

We conclude that real time observations of CCTV monitors, automated computer monitoring of CCTV signals, and/or training of drivers and transit personnel on behavioural indications of suicide risk, may identify attempters with few false positives, and potentially save lives.

## Background

In the Montreal underground urban transit system, the “metro”, there is an average of 15 suicide attempts each year. About two-thirds of suicide attempters do not die from their attempt but may be seriously injured. Besides the tragic loss of human life and injury, metro suicides are traumatic incidents for train drivers and first responders, as well as staff involved in cleanup and passengers who may witness the event. There are also substantial inconveniences for metro passengers and high costs for the Montreal urban transit system (STM). This article explores the possibility of identifying people on station platforms who are at risk of attempting suicide on the basis of observed behaviours, by observing video recordings from the metro closed circuit TV security surveillance system (CCTV) in stations, preceding their attempts.

One of the challenges in identifying the behaviours of future attempters is the issue of false positives. If behaviours of future attempters are also present in a substantial number of non-attempters, this could lead to unnecessary service disruptions and possibly intrusive interventions with people who are not at risk of attempting suicide. The overall objective of the project is to assess the feasibility of using live CCTV images to identify at risk people on platforms, based on their behaviours. Two studies were conducted to achieve this objective. Study 1 aimed at identifying the behaviours present in attempters on the metro property before their attempt that may be precursors of their attempt. The underlying hypothesis is that people in stations who are going to attempt suicide have recognizable behaviours that may be indications that a suicide attempt is imminent. The objective of study 2 was to assess the specificity of behaviour patterns that can be observed within five minutes of the attempt, by comparing videos of attempters with videos where no one attempted suicide. It is important to ascertain if the behaviours we observe in suicidal persons also occur in non-attempters on the platform, in order to determine the usefulness of using specific behaviours to identify people at risk of attempting suicide without significant numbers of false positives, that is, without also identifying significant numbers of non-attempters as “suicidal” because they have the same behaviours as the persons who will attempt suicide. In the second study, CCTV recordings of passenger behaviours in the stations when there was a future attempter present were randomly mixed with recordings of the same stations at the same time and day of the week, but when no future attempters were present. By asking many individuals to identify preselected behaviours in brief five minute recordings, we verified if it is possible to easily identify target behaviours in a timely manner, and if these behaviours are specific to suicide attempters, or are also observable in non-attempters. If the results are to have practical implications in preventing suicides, it is essential that suicidal individuals be identified rapidly in the minutes before an attempt, so that train drivers can be notified to slow their train and prepare to stop before entering the station.

Several research studies indicate that controlling access to a specific means of suicide saves lives, without significant substitution with other suicide methods [1–4]. Preventing access to the metro as a means to commit suicide generally involves installing barriers that inhibit access to the rails, but open to let passengers enter the train. Many new urban transit systems have barriers, but retrofitting barriers to many olders systems, which may be 50 to 100 years old, can be quite expensive and challenging. An alternative is to identify potential attempters based on their behaviours in stations prior to an attempt. There are few research investigations of the behaviours of suicide attempters on railway property with the purpose of preventing suicides. Most previous studies of behaviours prior to an attempt relied on recollections from train drivers or witnesses, whose reliability and validity may be questioned. Also, most existing data concern intercity and suburban railway systems, and not underground urban transit.

In Sweden, railway suicides do not often occur in stations; they are more often on open track. Radbo, Svedung and Andersson [5] found that most people (75%) who attempted suicide waited near the tracks or stood on the tracks. A small percentage (14.5%) jumped in front of the train from a platform or ran in front of the train. Another study [6] found that 36% walked along the rails and 30% laid down on the rail, and another 30% jumped or ran in front of the train. In Finland, half of suicide attempters waited for the train on the tracks and 18% jumped or ran in front of the train [7]. In the United States, 36% laid on the rails and 27% jumped in front of the train, and another 27% walked on the tracks. While these patterns may be of some interest, they give us little indication of identifiable behaviours before the attempt was initiated. Furthermore, results from studies of intercity railways may be difficult to apply to enclosed urban systems where tracks are only accessible from platforms.

In the United Kingdom, train drivers described behaviours they observed. However, these observations were generally when the train was less than 100 m from the attempter and it was often too late to stop the train [8]. A study in Germany involved interviewing 202 police officers about the behaviours of people who committed suicide on railways [9]. Although the police officers rarely observed the suicide attempt themselves, they obtained information from witnesses of the event and reported that 50% had left personal objects on the train platform, 50% avoided eye contact with others, 37% had behaviours or were talking in ways which witnesses considered “strange”, 25% seemed to be confused, and 15% seemed to be pacing around the platform “without purpose”. These data are to be regarded with caution, since witness recollections of events may not be reliable.

## First study: the identification of suicide Attempters’ behaviours in stations before their attempt

### Method

After obtaining approval from the Ethics Committee of the Université du Québec à Montréal, we obtained from the (Société de Transport de Montréal [STM] (2015). Internal Statistics. Unpublished) CCTV video recordings of all suicide attempts in the Montreal metro system from the 1st of January, 2010 to the 31st of December, 2013. We considered a suicide attempt to be any event in which an individual intentionally put him or herself in the path of a train, whether or not there was a collision, injuries or a death. Overall, there were 66 events that matched the criteria for suicide attempts during this period. The STM provided the research team with comprehensive recordings from all of the cameras in the station that captured images of the person before, during and after the attempt. In some instances, the same person was recorded simultaneously from cameras in several different positions with different points of view. Whenever possible, we observed the first sighting of the individuals when they entered the station to purchase tickets or pass through the entry turnstiles, until after the event when the person or the person’s body was taken out of the station. All videos provided in the first study contained a suicide attempt.

The amount of time each individual spent from first entering the station until their suicide attempt varies greatly, from a few minutes to several hours. Because of variations in the number of cameras and their placement in the stations and the different amounts of time the attempters spent in the station, the total length of recordings per incident varied from about one hour to over 20 h. All recordings were placed on a DVD and encoded so that only a person with a specific authorisation and password and a special decoding programme installed in their computer would be able to see the recordings.

First, one of the researchers (BM) watched all the recordings of a random sample of 14 incidents. The number of recordings initially observed was limited to 14 based upon the principle of data saturation: once no new categories of behaviours were observed after adding observations from four additional recordings, it was considered that a basis for preliminary categorization of behaviours was attained. Based upon his observations of those cases, he developed a preliminary list of categories to describe the person’s behaviours, which included all the behaviours previously mentioned in research and reports: leaving an object on the platform, anxious and depressive behaviours, seemed intoxicated, unusual or “strange” behaviours. The principal researcher then trained 4 doctoral students in psychology to watch the videos and identify all of the observed behaviours by the suicidal individual, including any additional behaviours which were present. An interrater agreement process was carried out to insure a high level of coding reliability. After a training session and collective coding of a case, the research assistants watched videos at the same time and noted all observed behaviours independently. They then compared their observations and discussed any differences in order to refine definitions and obtain a common understanding of the behaviour categories. Training was scheduled to continue until there was complete agreement between raters. However, there were no disagreements in categorisation in the pre-established categories between the research assistants after the initial training period. The principal investigator also compared his observations on the 14 cases he coded with the observations by the research assistants on the same cases and watched an additional five cases to verify the reliability of their observations. Agreement between raters was total. There were, however, some instances where one of the two observers included a behaviour which was not part of the original list and which the other had not included. These additional observations were compiled in our descriptions of the behaviours in the station. All videos were rated independently by two research assistants in order to validate coding and make sure that all observed behaviours were recorded.

The four people who made the ratings were recruited by an announcement among the graduate students associated with our research centre. Originally, there were six individuals who volunteered to work on the project. However, when first starting their work, two of the six found it too disturbing to watch these videos and discontinued working on the project. The four observers who remained did not report any problems or negative consequences from watching the videos. Still, they were given frequent opportunities for debriefing and were offered help. However, all said that they felt there were no negative reactions to their work on this project.

### Results

**Fig. 1:**
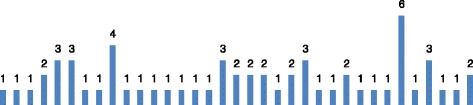
Number of suicide attempts by station

**Fig. 2:**
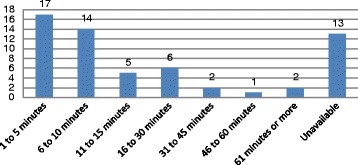
Time between entering the station and the suicide attempt

Six of the 66 CCTV recordings had a substantial portion of the recordings that was not of sufficient quality to be used for analyses. Therefore, our results concern 60 cases, 41 men, 18 women and one case where we were not able to identify the sex of the victim from the recording. The incidents occurred in 35 of the 68 metro stations with several stations having more than one attempt (see Fig. 1). Most attempts (*N* = 36, 66%) occurred in the first 15 min after the person entered the station, and often (*N* = 31, 52%) in the first 10 min after entering the station. Therefore, there is generally relatively little time to identify a person on the platform before intervening (see Fig. 2).

### Behaviours when entering the station

In 47 of the 60 cases (78%), we have CCTV recordings of the persons as they entered the station and in eight instances, the persons arrived on the platform where they attempted suicide from another station. Of the 47 for whom we have recordings of their entering the station, 11 (23%) purchased a ticket, 19 (40%) already had a ticket or pass in their possession, and nine (19%) entered without paying by going over the barriers illegally.

In most cases, there appeared to be nothing unusual about their behaviours upon entering the stations. However, in seven instances (15%), they appeared to have some difficulty when purchasing their ticket or when validating their ticket at the turnstile. Two people (4%) seemed disoriented and confused, four (7%) seemed agitated or nervous, and nine (19%) talked to someone (three talked to an employee, two to another passenger, and three talked on their cellular (portable) telephone). There was only one case (2%) where the person was accompanied when entering the station (in this case, the woman attempted suicide and the man who accompanied her unsuccessfully tried to stop her during her attempt).

### Behaviours on the station platform

**Fig. 3:**
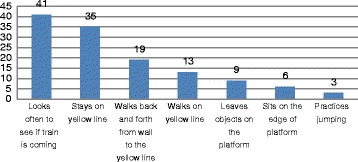
Frequency of occurrence of easily observable behaviours by suicide attempters

**Fig. 4:**
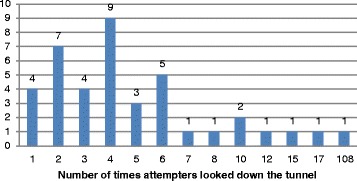
Frequency of looking down the tunnel

**Fig. 5:**
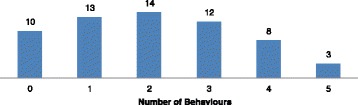
Total Number of Easily Observable Behaviours Present for Each Suicide Attempter

**Fig. 6:**
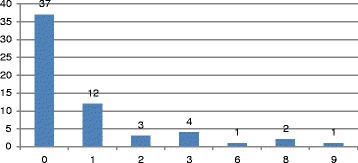
Total number of trains that passed before each suicide attempt

There were two categories of behaviours on the station platform: behaviours whose presence or absence could be reliably observed without interpretation and behaviours which required some interpretation. Behaviours which we felt did not need to be interpreted were analysed separately because it was thought that it may be possible to develop a computer programme to detect these types of behaviours. Fig. 3 shows the frequency of occurrence of easily observable behaviours. A majority (*N* = 41, 68%) frequently looked down the tunnel for the train to arrive (see Fig. 4). The total number of times people looked down the tunnel varied from once in four instances, to one person who looked 108 times. Nine people (15%) left an object on the platform and 48 (80%) either were walking on the yellow line (which passengers are not supposed to cross until the train arrives), or stood on the yellow line. Pacing back and forth to and from the yellow line was also fairly frequent (*N* = 19, 32%). Three people (5%) seemed to practice jumping and six (10%) sat on the edge of the platform. Overall, the vast majority had some observable behaviour (see Fig. 5). One-third had three or more of these behaviours (*N* = 23, 38%) and (67%) had a behaviour involving crossing or standing on the yellow line. In addition, 24 (40%) waited on the platform until one or more trains passed before attempting suicide, and 11 (18%) waited for at least two trains to pass. No other persons on the platform did so in these 60 observations (see Fig. 6).

### Observed behaviours which require interpretation

**Fig. 7:**
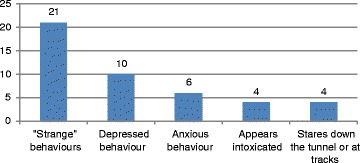
Frequency of occurrence of behaviours requiring interpretation

The frequencies of observed behaviours which require interpretation are shown in Fig. 7. The 21 instances (35%) of “strange behaviours” were behaviours which appeared to be symptomatic of someone with a mental health problem or intoxicated, such as odd gestures, talking to oneself, or having motions which appeared to be out of place. In all instances, these behaviours were validated by two independent observers. Of the 16 cases (27%) of behaviours indicating anxiety or depression, 10 (17%) appeared depressed because of their physical behaviour, such as hunching over and moving slowly, and six (10%) appeared anxious. Only four (7%) appeared to be intoxicated and another four (7%) stood looking steadily either down the tunnel or down at the rails. Few talked with others, but we did observe ten (17%) persons talking with another passenger, three (5%) talking on their cellular (portable) telephone, and two (3%) talking to a metro employee. In one of those instances, the person descended onto the rails, the employee talked to the person, who then came back up on the platform, and when the employee went away (presumably to get help), the person went down again onto the rails and committed suicide by electrocution. Since we did not have audio recordings, we have no information about the nature of any of the conversations.

**Fig. 8:**
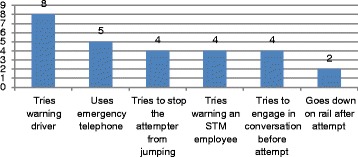
Incidents when witnesses tried helping the attempter

In 22 instances (37%), witnesses to the event attempted to save the person who was attempting suicide (see Fig. 8). In several instances, they tried to get help or physically stop the person. In 19 instances (32%), they came closer to see what had happened after the attempt, and 11 (18%) of the witnesses talked to another person after the event.

**Fig. 9:**
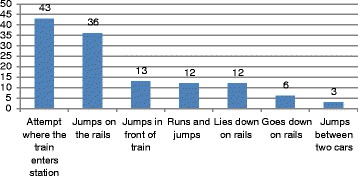
Manner of suicide attempt

The manner of the suicide attempt is described in Fig. 9. Over two-thirds (*N* = 43, 72%) occurred at the beginning of the station, where the train arrives. Thirty-six (60%) jumped down onto the rails and another 13 (22%) jumped right in front of the train (they were often hit by the train before touching the ground). Six (10%) descended from the platform to stand on the tracks and 12 (20%) lay down on the rails. In 12 (20%) instances, they ran and jumped and in three instances (5%), they jumped between two cars (it is not clear if this was intentional).

### Indications of possible ambivalence

**Fig. 10:**
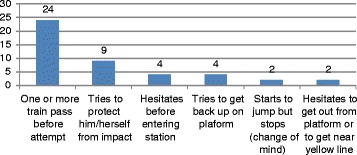
Indications of possible ambivalence

We observed possible signs of ambivalence toward suicide in the behaviours of a number of attempters. One possible indication of ambivalence is the large number of persons (*N* = 23, 38%) who waited for one of more trains to pass before initiating their attempt. We observed several other possible indications of ambivalence. Four (7%) of the persons who attempted suicide hesitated for a long time before passing through the turnstile to enter the station. In one instance, the person hesitated before buying the ticket. He then entered the station and stopped immediately after passing the turnstile, hesitated, and left through the turnstile. He stopped and hesitated, talked to the ticket seller, and then went back in through the turnstile, down to the platform, where he waited until a first train passed before attempting suicide with the next train. In four instances (7%), the person who went down on the tracks tried unsuccessfully to get back onto the platform before impact from the train. Nine (15%) of the people who were on the tracks appeared to try to protect themselves from the impact of the train by ducking their head and body down as close to the ground as possible as the train arrived. Two (3%) began to jump and tried unsuccessfully to stop during their jump, and two (3%) others started to leave the station from the platform, then turned around and came back. Overall, 45 persons (75%) showed some signs of ambivalence or indications that they at least temporarily had changed their mind (see Fig. 10).

## Second study: assessment of the efficacy of real-time analysis of behavioural patterns to identify suicide attempters on metro platforms

### Objectives

The second study had two objectives: The first was to verify the ability of the behaviour patterns identified in the first study to discriminate suicidal persons from other passengers on the metro platform, that is, the presence of false positives. The second was to verify the feasibility of using live observations of peoples’ behaviours captured by CCTV to rapidly and reliably identify suicidal persons before they attempt. If these findings are to be of practical use, the identification of suicide attempters must be made rapidly in the minutes before the train arrives in the station, so that the driver may be notified in time to slow the train and prepare to stop, with relatively few false positive results. In our first study, research assistants watched up to 20 h of videos from multiple CCTV cameras and were able to take their time in identifying target behaviours. They could rewind and watch scenes as often as they needed in order to make sure that they correctly identified behaviours. However, in real life situations, assessments would have to be made within minutes in order to immediately alert train drivers and intervene.

## Methods

### Recordings

We obtained recordings from a single camera showing the segment of platform where a suicide attempter was located in the 5 minutes preceding the attempt for all the suicide attempts assessed in the first study. We also obtained 5 minutes recordings of the same platform, at the same time of day and the same day of week from the same camera, but on a day when no suicide attempts occurred in that station. None of the recordings continued up to the point when an attempt was initiated. Overall, we obtained a total of 132 5-minute videos, 66 from situations where there was a suicide attempter on the platform and 66 where no suicide attempter was present. We eliminated 10 videos in the control situation, where there was no one seen on the platform by that CCTV camera during the 5-min segment. We eliminated two in the experimental condition videos, one where the suicidal person was only seen by the camera for a short period of time, and another where the image was not sufficiently clear to see what was occurring. The final sample included 56 recordings without a suicide attempter in the station and 63 when there was a person in the station who was going to attempt suicide there. These 5-min segments were randomly ordered and divided into four three hour sessions, each with 29 or 30 of the 5-min videos.

### Instruments

A coding sheet was designed to allow for real-time assessments of the presence of all the behaviours we observed in Study 1. It includes the list of behaviours, a brief description of what is meant by that category and a space to note any further observation or comments about the observation of that behaviour.

### Raters

We recruited 33 students registered in the Bachelor of Psychology programme at UQAM. who were paid to watch the video recordings and indicate on a coding sheet if they observed each of the behaviours listed on the sheet in any person they saw in each video. Their goal was not to identify people who were suicidal or not suicidal, but simply to indicate if anyone seen in the 5-min segment engaged in each of the target behaviours. A ten minutes training session explained that the ultimate goal was to identify people at risk of attempting suicide, but we clearly indicated that there may or may not be one or more people with these target behaviours in each of the videos, and they would not be watching any suicide attempts. The coding sheet for the targeted behaviours was explained and questions were answered before coding started. The students were told that they were to watch each segment and immediately check on a coding sheet if there was anyone who engaged in each of the behaviours listed. We intentionally did not give detailed training in order to see if someone with little training and just a list of behaviours to observe is capable of identifying people who are at risk of suicide in real time, while watching CCTV surveillance monitors. This procedure was designed to reflect some of the conditions in which real life assessments could be made (naïve observers, short time span for assessment, and no rewinding of the images to watch them again).

### Procedure

The videos were projected on a screen in a classroom. While watching each segment and immediately afterwards, the raters checked on the sheet which of the behaviours, if any, were present in the video they watched (see Table 1). Afterwards, they were encouraged to describe their observations on the sheets. The rating sheets were then collected and the next 5-min video was shown and rated on a separate rating sheet. The four coding sessions took place over a week, and were attended by between 19 and 23 raters.

**Table 1:** List of behaviours observed in CCTV recordings

Sits on the edge of the platform	
Seems to practice jumping	
Leaves objects on the platform	
Often looks down the tunnel	
Walks back and forth between the wall and the yellow line	
Paces on the yellow line	
Stands on the yellow line for a long time	
Strange behaviours	
Anxious or depressive behaviours	
Seems intoxicated	
Stares at the tracks or down the tunnel for a long time	
Psychomotor agitation (repetitive behaviours, nervousness)	
Looks glum (shoulders hunched, head lowered, looks at the ground)	

### Data analysis

We considered a behaviour to not be present when none of the raters observed that behaviour in that video or when only one or two persons indicated that that behaviour was present. Our analyses revealed that there were four individuals who tended to almost always rate that there was someone who seemed anxious or depressed in all the videos, as well as someone with strange behaviours. In coding observations in real time, one would assume that people who always rate infrequent behaviours as always being present would not be selected to identify the rare instances of having a suicide attempter in the station. We did not pre-screen the volunteer observers for their ability to rate the videos. However, because of their extreme deviation from the ratings of all the other observers, who identified very low frequencies of each behaviour, we eliminated all the ratings by these four people.

## Results

### Interrater agreement

We calculated Fleiss’s Kappa, a statistical measure for assessing the inter-rater reliability among multiple raters using binary or nominal scale ratings, for the behaviour ratings. The behaviours which we felt require interpretation had lower inter-rater reliability: Anxious or Depressive Behaviour had “slight agreement,” (Kappa .0783; *SE* = .0060), Seems Glum (Kappa .2389; *SE* = .0088) and Psychomotor Agitation (Kappa .2715; *SE* = .0059) had “fair agreement”, and Strange Behaviours had “moderate agreement” (Kappa .4107, *SE* = .0061). However, the behaviours which we felt did not need to be interpreted has much higher inter-rater reliability: “Substantial agreement” was found for Looks Frequently Down the Tunnel (Kappa .6488, *SE* = .0024) and Walks Back and Forth to the Yellow Line (Kappa .6063, *SE* = .0072), and there was “almost perfect agreement” for Leaves an Object on the Platform (Kappa .8417, *SE* = .0059).

### Behaviours observed

In the 63 instances where there was a person on the platform who was going to attempt suicide, none of the target behaviours were observed in almost half the cases (*n* = 30, 47%). Among the 53% who had identifiable behaviours, several behaviours were only present in suicide attempters and never observed in any of the recordings when no suicide attempter was on the platform. Five of the attempters left an object on the platform (8%) and this was statistically significantly different from the videos without attempters (*χ* ^2^ = 4.29, *df* = 1, *p* < .04). This behaviour was accompanied by other target behaviours in all instances: one of the five also paced back and forth between the yellow line and the wall, one also had psychomotor agitation, a third was also both agitated and often looked down the tunnel, and the remaining two also paced back and forth between the yellow line and the wall, spent long periods of time standing on the yellow line, stared for long periods of time at the rails and had psychomotor agitation (see Table 2).

**Table 2:** Behaviours observed in future suicide attempters (*n* = 63)

Behaviour	Combined Behaviour(s)	*n*
Nothing observed	--	30
Leaves an object on the platform	Walks back and forth between the wall and the yellow line	1
	Psychomotor agitation	1
	Psychomotor agitation and looks frequently down the tunnel	1
	Walks back and forth between the wall and the yellow line, stands on the yellow line and stares at the tracks	2
	Total	5
Walks back and forth between the wall and the yellow line	--	2
	Leaves an object on the platform	2
	Psychomotor agitation	1
	Seems to practice jumping	1
	Looks frequently down the tunnel and stares at the tracks or down the tunnel for a long time	2
	Stays on the yellow line	1
	Stays on the yellow line, stares at the tracks and seems anxious and agitated	1
	Stares at the tracks for a long time, psychomotor agitation and « strange » behaviours	1
	Often looks down the tunnel, walks often on the yellow line, « strange » behaviours and psychomotor agitation	1
	Total	12
Looks glum	--	3
	Paces on the yellow line, stares at the tracks and seems agitated	2
	Total	5
Stands on the yellow line for a long time	--	2
Looks frequently down the tunnel	--	2
	Stares at the tracks	1
	Stays on the yellow line and stares at the tracks	1
	Stays on the yellow line and psychomotor agitation	3
	Paces on the yellow line	1
	Paces on the yellow line and stares down the tunnel	1
	Total	9
Walks repetitively on the yellow line	Stares at the tracks and psychomotor agitation	2

A second behaviour observed only in persons who were going to attempt suicide was to pace back and forth between the yellow line and the wall, which was present in 12 individuals. Two of the 12 were already mentioned as having left an object on the platform, one also had psychomotor agitation, one also seemed to practice jumping, two also looked frequently down the tunnel, one stood on the yellow line and one stood on the yellow line, watched the rails for long periods of time and had psychomotor agitation, and two others had a combination of several other behaviours (see Table 2).

Five of the attempters (8%) seemed depressed (shoulders hunched, head lowered, looking at the ground, etc.). This compares with only one person who was not to attempt suicide and the difference between attempters and non-attempters was not statistically significant. Two of these attempters who seemed depressed also had other behaviours of pacing back and forth on the yellow line, regarding the tracks and psychomotor agitation.

The remaining 13 (20%) of people who were to attempt suicide had behaviours which were also observed in non-suicidal individuals. Again, in the case of attempters, these behaviours were generally accompanied by two or more other observed behaviours, which was not the case with people who were not going to attempt suicide. Frequently looking down the tunnel was present in 10 attempters (16%), but also one non-attempter; the difference was statistically significant (*χ* ^*2*^ = 7.01, *df* = 1, *p* < .006). However, only in the case of people who were to attempt suicide, this behaviour was often accompanied by other behaviours. Only two (20%) of the 10 had the behaviour of frequently looking down the tunnel unaccompanied by other behaviours (see Table 2).

Among the 56 videos where there was no person who was going to attempt suicide on the platform, in 10 instances (18%), one or more of the target behaviours was observed. Two of these (4%) were people who seemed intoxicated, one sat on the edge of the platform, five (9%) stayed on the yellow line combined with either frequently looking down the tunnel and seeming depressed in one instance or with staring down the tracks or down the tunnel for long periods of time (see Table 3).

**Table 3:** Behaviours observed in Non-suicidal persons (*n* = 56 cases)

Behaviour(s)	n
Nothing observed	46
Seems intoxicated and psychomotor agitation	2
Sits on the edge of the platform	1
Stares at the rail or down the tunnel for a long time and looks glum	2
Stands on the yellow line for a long time, frequently looks down the tunnel and looks glum	1
Stands a long time on the yellow line and stares at the rail or down the tunnel for a long time	4
Total	56

## Discussion

Study 1 provides a description of the behaviours of people before they attempt suicide. We were able to identify a number of behaviours on the platform that could be considered indications of risk of an impending suicide attempt. Some of these behaviours are specific to persons who are going to attempt suicide. When more than one of these behaviours is present, their usefulness for identifying future attempters is improved. In Study 1, the behaviours we observed in persons who were going to attempt suicide were not present in other individuals in the same stations at that time. These behaviours may reflect ambivalence, anxiety or planning before the suicidal act. According to theories and practical experience in suicide prevention, most people who attempt suicide experience ambivalence before and at the time of their attempt, and many change their mind after initiating an attempt and call emergency services or a crisis line, and often stop their attempt if they can [10]. We observed a number of possible indications of ambivalence in three-quarters of attempters, including hesitation and trying to avoid being hit after having placed oneself in front of the train.

Some behaviour may be easier to observe by watching videos or using an automated surveillance system, such as leaving an object and walking back and forth to the yellow line. Other behaviours are more complex and require a certain level of analysis, such as determining if a person “seems depressed.” The behaviours that require interpretation may be reliably identified by intensively trained observers, as shown in Study 1. However, Study 2 shows that although minimally trained observers can reliably identify the easily observable behaviours, their inter-rater reliability is only fair for behaviours involving interpretation.

Study 2 showed us that many of the behaviours identified in Study 1 were easily observed in suicidal persons on the platform in the 5 minutes before their attempt. The fact that the second study was limited to observations from a single camera during the 5 minutes before the attempts, rather than all the cameras and the entire time the suicidal person was in the station, led to us only observing the target behaviours in half (53%) of the cases. However, some behaviours, such as staying on the yellow line and frequently looking down the tunnel or staring at the rails, may also be observed in people who are not going to attempt suicide, even if these behaviours may be present more often in suicidal individuals. The identification of someone who “seems depressed” does not appear to be a selective indicator that can be used to identify suicidal individuals. However, when seeming depressed occurred in combination with other behaviours, this was unique to attempters.

Based on the results, 24% of future attempters can be identified, without identifying any non-suicidal persons as being at risk by the presence of either of two behaviours: leaving an object on the platform and pacing back and forth between the yellow line and the wall. Other behaviours that were observed more frequently in persons who are going to attempt suicide were also observed in some non-suicidal individuals. However, in the case of people who are to attempt suicide, these behaviours are almost always present in combination with other behaviours. Therefore, the presence of two or more of the following behaviours may indicate risk of a suicide attempt: frequently looking down the tunnel, standing for long periods of time on the yellow line, continually walking on the yellow line, psychomotor agitation, staring at the tracks or the tunnel for long periods of time, bizarre behaviours and seeming depressed.

Several behaviours we observed may be able to be identified automatically by sophisticated computer programmes that analyse CCTV images in real time. Also, it may be possible for train drivers to recognize these behaviours, particularly if they have monitors in their trains to see the passengers on the platform before the train enters the station. Even if some of the behaviours, such as frequently looking down the tunnel, may be also be present in some non-suicidal individuals, being vigilant and slowing down the train may still be warranted when they are observed. Also, other railway personnel, such as people doing maintenance or cleaning in the stations, may benefit from training to identify these behaviours.

It is important to note that not all future attempters had observable indications that they were at risk of attempting suicide. The only completely effective strategy is to install automatic barriers with doors that open only when the train is in the station, that make it impossible for passengers to have access to the tracks. This structural intervention exists in several urban transit networks and was proven to be effective in decreasing suicides in a study conducted in Hong Kong over 11 years [11]. There was no indication in the study that suicides increased in stations which were not equipped with these automatic door systems.

The one factor identified in the first study which was not validated in the second study, because of using only 5-min videos before the train arrived, was waiting on the platform until one or more trains passed. Casual observations in the metro indicate that this does sometimes occur in people who are not to attempt suicide, but the frequency of this behaviour is not known. It still may be a useful to observe persons who wait for more than one train to pass, to see if other behaviours are present.

Because of the risk of false positives, it is important in future surveillance efforts to validate that the behaviours identified have the ability to discriminate attempters from non-attempters. For example, people who seem drunk or depressed may sometimes be present in stations. Although these behaviour patterns may be more frequent in attempters, their discriminating power may not be sufficient to warrant their use.

## Conclusions

People who attempt suicide in the metro have specific behaviour patterns before their attempt that may differentiate them from people who are not going to attempt suicide. Some suicides in urban transit systems may be prevented by identifying people exhibiting these behaviours in the station, either through observations by employees working in the station, train drivers, or real time monitoring of CCTV videos. It may also be possible to develop automated computer programmes to conduct real time analyses of videos, and either signal train drivers to use caution or slow down before approaching the station, or at least signal that someone should immediately look at a specific camera monitor to see if a person appears to be at risk.

Obviously, interventions just before a person is about to attempt suicide do not prevent people from becoming suicidal nor from going to the metro to attempt suicide. For this reason, it is important to also engage in more global local and national suicide prevention programmes to prevent people from becoming suicidal and from choosing the metro as a means to commit suicide.

## Abbreviations

CCTV: Closed Circuit Television
STM: Société de transport de Montréal (The public transportation corporation for Montreal)
UQAM: Université du Québec à Montréal
